# Especialização em análise multivariada: uma contribuição brasileira à saúde pública e além 

**DOI:** 10.1590/0102-311XPT211723

**Published:** 2024-02-19

**Authors:** Marcelo L. D. S. Gabriel

**Affiliations:** 1 Escola Superior de Propaganda e Marketing, São Paulo, Brasil.

Por muitos anos, esperamos uma obra sobre análise multivariada de dados, cuja aplicação é recorrente nas pesquisas em Saúde Pública, particularmente, e nas Ciências Humanas e Sociais em geral, escrita em português e por autores brasileiros que, como professores da disciplina, conhecem e reconhecem as lacunas na formação dos alunos e futuros pesquisadores.

As obras até então disponíveis, tanto para os aprendizes do tema quanto para os pesquisadores mais maduros, limitavam-se a traduções em português de livros-texto em inglês ^1^^,^^2^, ou a leituras diretas de livros escritos em inglês ^3^^,^^4^^,^^5^, adicionando mais uma camada de complexidade ao aprendizado.

Na obra de Artes & Barroso ^6^, recentemente publicada, encontramos um conjunto de métodos e técnicas de análise estatística multivariada que não esgota a totalidade das possibilidades, mas releva aquelas mais comumente aplicadas nas pesquisas.

Ainda que escrito originalmente em nosso idioma, usa uma linguagem bastante técnica, redigida por estatísticos e endereçada a leitores com conhecimento prévio de estatística descritiva e inferencial, cálculo e álgebra linear, conceitos fundamentais para a compreensão das diferentes derivações feitas ao longo dos capítulos, recheadas de equações e pressuposições *a priori*.

Logo no início do livro, o leitor é apresentado às notações e aos resultados básicos da análise multivariada de dados, como matrizes de dados a partir de bases, distribuição bivariada e multivariada que, como já mencionado, requerem minimamente o domínio prévio dos conceitos.

O capítulo seguinte, intitulado *Estatística Descritiva*, conduz o leitor a uma imersão nos conceitos explicados superficialmente nos cursos introdutórios de estatística descritiva e inferencial, tanto no âmbito da graduação quanto da pós-graduação.

Ao propor essa jornada às razões subjacentes das análises, os autores se apoiam novamente em equações e exposição dos passos necessários a cada etapa. Nesse ponto, os leitores menos familiarizados com os conceitos considerados como pré-requisitos à leitura podem se sentir motivados a pular alguns parágrafos.

Diferentemente das obras mais utilizadas no ensino e aprendizagem da análise multivariada de dados até hoje, os autores privilegiam a especificidade de cada método ou técnica em capítulos específicos, como é o caso da análise de componentes principais (ACP) que usualmente é apresentada em conjunto com a análise fatorial exploratória (AFE) nos livros-texto atuais, o que leva a uma sobrecarga das considerações sobre a AFE em detrimento da ACP.

Apesar do uso da linguagem R (http://www.r-project.org) como base para todas as análises - que, senão por outro motivo, é justificado pela sua gratuidade -, no capítulo relativo à ACP, todos os passos são feitos a partir dos conceitos explicados, sem a utilização de pacotes específicos, como o *psych* (https://cran.r-project.org/package=psych) ou o *FactoMineR* (https://cran.r-project.org/package=FactoMineR), que adicionariam uma terceira camada de complexidade aos leitores que não têm a base teórico-conceitual necessária.

O mesmo efeito não se nota no capítulo relativo à AFE, em que se emprega o pacote *psych* na sintaxe utilizada. A escolha do número de fatores a ser retido em uma AFE têm sido amplamente discutida na literatura ^7^, com partidários do critério de normalização de Kaiser (autovalores acima de 1,0) e adeptos da análise paralela apresentando suas razões, os prós e os contras de cada abordagem, algo que não é abordado quando o tema é mencionado.

Se a distinção entre ACP e AFE é explorada em capítulos distintos, esse cuidado não se dá com a análise fatorial confirmatória (AFC), que é abordada dentro do capítulo sobre AFE em três parágrafos.

A inclusão da correlação policórica como abordagem apropriada para dados obtidos por meio de variáveis ordinais ^8^, como os coletados por escalas de mensuração do tipo Likert, fornece ao leitor um novo caminho para interpretar e analisar os resultados, raramente encontrado nos livros-texto disponíveis atualmente.

Seguem-se capítulos sobre escalonamento multidimensional (EM), análise de correspondência (AC), análise de correlação canônica (ACC), análise de agrupamentos (AA) e análise discriminante (AD). Para a AC, é utilizado o pacote *ca* do R (https://cran.r-project.org/package=ca), bem como o pacote *cca* (https://cran.r-project.org/package=cca) para a ACC, o pacote *cluster* (https://cran.r-project.org/package=cluster) para a AA, e o pacote MASS (https://cran.r-project.org/package=MASS) para a AD e o EM, o que pode facilitar a replicação das análises dos leitores com seus próprios dados.

Em tempos de ciência de dados (*data science*) e *big data*, o livro apresenta um capítulo sobre árvores de decisão (ADec), com base em análise de regressão, um método pouco explorado na literatura atual em análise multivariada de dados, mas onipresente nos livros-texto sobre mineração de dados, tanto em aplicações supervisionadas como *classification and regression trees* (CART) quanto em aplicações não supervisionadas como o *chi-square automatic interaction detection* (CHAID).

A inclusão desse método como um capítulo aponta em direção a uma ampliação do instrumental necessário ao pesquisador em Saúde Pública (e nas Ciências Humanas e Sociais) para identificar padrões nos dados, não previamente identificados pelo método hipotético-dedutivo, utilizando grandes bases e pesquisa baseada em dados (*data driven research*).

Apresentado como um apêndice, os autores desenvolvem um capítulo sobre construção e validação de escalas, apenas com a abordagem da teoria clássica dos testes (TCT) e nenhuma menção à teoria de resposta ao item (TRI), que vem sendo empregada há muitos anos nos exames realizados pelo Instituto Nacional de Estudos e Pesquisas Educacionais Anisio Teixeira (Inep), como o *Exame Nacional do Ensino Médio* (Enem). Ainda que citem autores clássicos e consagrados no campo da psicometria, para uma obra atual seria importante revisar as referências visto que a maioria das obras citadas está uma ou duas edições desatualizadas.

Com o avanço dos métodos multivariados a partir da disponibilidade computacional e das aplicações estatísticas, mormente a linguagem de programação R, um capítulo que poderia ser incorporado em uma segunda edição do livro seria sobre análise de caminhos (do inglês *path analysis*), AFC com maior detalhamento, combinando com ACC e, como consequência, a modelagem de equações estruturais (MEE), que não é um tema desconhecido para pelo menos um dos autores.

Todavia, ainda carecemos de uma obra contemporânea sobre a MEE, escrita em português, por autores que conheçam o tema e possam remover as barreiras técnicas e linguísticas do aprendizado de um método analítico de segunda geração.

Considerado no todo, o livro vem preencher uma lacuna em nossas referências nacionais, mas seu alto grau de complexidade e pressuposições sobre os conhecimentos prévios dos leitores não atende à política editorial declarada pela Associação Brasileira de Estatística (ABE) em uma das páginas iniciais, cujo foco pretende estar nos alunos do bacharelado. Uma nova edição poderia fornecer algumas sugestões para avaliação *a posteriori* dos resultados, pois ao remeter o leitor às obras de referência em inglês, retoma a problemática introduzida no início desta resenha, com suas camadas de complexidade.
